# In Vivo-In Vitro Comparative Toxicology of Cadmium Sulphide Quantum Dots in the Model Organism *Saccharomyces cerevisiae*

**DOI:** 10.3390/nano9040512

**Published:** 2019-04-02

**Authors:** Luca Pagano, Marina Caldara, Marco Villani, Andrea Zappettini, Nelson Marmiroli, Marta Marmiroli

**Affiliations:** 1 Department of Chemistry, Life Sciences and Environmental Sustainability, University of Parma, 43123 Parma, Italy; luca.pagano@unipr.it (L.P.); marina.caldara@unipr.it (M.C.); nelson.marmiroli@unipr.it (N.M.); 2 IMEM-CNR, 43123 Parma, Italy; marco.villani@imem.cnr.it (M.V.); andrea.zappettini@imem.cnr.it (A.Z.); 3 Consorzio Interuniversitario Nazionale per le Scienze Ambientali (CINSA), University of Parma, 43123 Parma, Italy

**Keywords:** comparative toxicology, transcriptomics, phenomics, gene ontology, quantum dots

## Abstract

The aim of this work was to use the yeast *Saccharomyces cerevisiae* as a tool for toxicogenomic studies of Engineered Nanomaterials (ENMs) risk assessment, in particular focusing on cadmium based quantum dots (CdS QDs). This model has been exploited for its peculiar features: a short replication time, growth on both fermentable and oxidizable carbon sources, and for the contextual availability of genome wide information in the form of genetic maps, DNA microarray, and collections of barcoded mutants. The comparison of the whole genome analysis with the microarray experiments (99.9% coverage) and with the phenotypic analysis of 4688 barcoded haploid mutants (80.2% coverage), shed light on the genes involved in the response to CdS QDs, both in vivo and in vitro. The results have clarified the mechanisms involved in the exposure to CdS QDs, and whether these ENMs and Cd^2+^ exploited different pathways of response, in particular related to oxidative stress and to the maintenance of mitochondrial integrity and function. *Saccharomyces cerevisiae* remains a versatile and robust alternative for organismal toxicological studies, with a high level of heuristic insights into the toxicology of more complex eukaryotes, including mammals.

## 1. Introduction

Engineered nanomaterials (ENMs) have a nanoscale level range of 1–100 nm, whose surface area can cause a higher reactivity. Some ENMs may show peculiar physico-chemical properties (optic, magnetic, dielectric, of density and mechanic resistance) and for those reasons are currently used in different areas such as electronics, biomedicine, pharmaceuticals, cosmetics, environmental analysis and remediation, catalysis and material sciences [1]. Their market size has been approximately estimated to be 55 billion USD by 2022 [2,3]. There are so many nanomaterials-enabled products that a recent simulated LCA (life cycle analysis) shows that there is potential for an increasing amount of ENMs that could reach aquifers, soil and air in the near future, through processes such as product waste disposal [4]. Some potential toxicological effects of ENMs on human health and to a lesser extent on the environment have been elucidated, although there is still an insufficient degree of understanding of the mechanisms of uptake and the nature of the cellular processes involved [5,6,7]. Their large use, especially in medicine, and their possible environmental dispersion raise two important questions: the health effects consequent to the potential exposure to humans and the possible environmental damage. For the health assessment, both epidemiological and analytical tools have been developed and tested [5,6]. For the environmental effects, there are many genetic reports of how ENMs can be monitored and measured [7,8,9]. These studies involve both food chains and ecosystems analyses. Clearly there is a need for a paradigmatic shift in nanotoxicology that enhances the use of alternative testing strategies (ATS), as advocated in 2007 by the National Academies of Sciences [10]. Among the more common categories of ENMs on the market, cadmium sulphide quantum dots (CdS QDs), due to their size/volume ratio and their dielectric and magnetic properties, have been relevantly applied to components of solar panels, new generation LED screens and lasers [11,12,13]. With the aim to investigate, at the transcriptomic level, the mechanisms involved in the CdS QDs response, the unicellular eukaryote *Saccharomyces cerevisiae*, one of the most used model organisms for molecular biology [14], has been chosen. Due to its easy use, short life cycle and availability of several molecular and genomic tools, yeast has become a platform for fast toxicological studies using high throughput techniques [15,16]. Furthermore, the high level of functional conservation between yeast genes and their orthologs in higher eukaryotes, including human orthologs, makes yeast a useful system to assess the toxicological mechanisms underlying the response to a wide range of ENMs [16,17,18,19]. In recent years, this model organism allowed elucidating the phenomic implications related to CdS QDs exposure, revealing the complex networks of interaction in which the genes *HSC82*, *ALD3* and *DSK2* played key roles [16]. Moreover, Pasquali et al. (2017) [20] suggested a connection between CdS QDs’ toxicity, mitochondria organization and functions. The preservation of this response has also been retrieved in human cells (HepG2) in which the stress caused by CdS QDs increases the production of reactive oxygen species (ROS), and is able to trigger the mitochondria-mediated intrinsic apoptotic pathway, which involves genes related to apoptosis, oxidative stress response and autophagy [21]. Other studies on baker’s yeast demonstrated an intrinsic instability of copper oxide (CuO) ENMs, enough to attribute its toxicity to Cu^2+^ ions release [17,22], while also demonstrating that the functionalization of Au ENMs, until now considered non-toxic, indeed affects the respiratory metabolism [23]. One of the greatest advantage of baker’s yeast is the possibility of a more general toxicological analysis, focused on the effects of substances or conditions upon the expression of specific cloned human genes potentially involved in fundamental diseases, what is called a humanized yeast model [24,25]. In this work the phenotypic response of *Saccharomyces cerevisiae* to CdS QDs mutant lines was compared with a high throughput microarray to investigate the genes involved in the response, molecular features of their involvement, and the biological pathways under their control. A comparison with the results obtained in higher eukaryotes including human cells was consistent with yeast as a toxicological model [21].

## 2. Materials and Methods

### 2.1. Cadmium Sulfide Quantum Dots (CdS QDs) Characterization

The uncoated CdS QDs that were used had a bulk density of 4.82 g·cm^−3^ and a mean diameter of 5 nm [16,26]. QDs were manufactured by IMEM-CNR (Parma, Italy), following the method described by Villani et al. (2012) [27]. QDs in deionized water were characterized by transmission electron microscopy (TEM) (Hitachi HT7700, Hitachi High Technologies America, Pleasanton, CA, USA) [16,20]. The average particle size of the aggregates (d_h_) and zeta (ζ) potential, respectively of 196.0 nm and +15.2 mV, were estimated in deionized water by Zetasizer Nano Series ZS90 (Malvern Instruments, Malvern, UK), as reported in Pagano et al. (2017) [28].

### 2.2. Strains Used

The *Saccharomyces cerevisiae* strain BY4742 (MATα), which contains the mutations *his3Δ1, leu2Δ0*, *lys2Δ0* and *ura3Δ0* was used for microarray experiments, Real Time PCR validation tests and as a control in the physiological tests considered. In the physiological test, the knock-out mutants (EUROSCARF) sensitive *ymr276w* (*dsk2*), *ymr169c (ald3*), *ypr133w-a* (*tom5)*, and tolerant *ylr342w* (*fks1*), *yol091w* (*spo21*), *yil123w* (*sim1*) to CdS QDs, respectively (as defined in Marmiroli et al., 2016 [16]), were employed.

### 2.3. Microarray Experiments

The BY4742 strain was grown in YPD (1% *w*/*v* Yeast extract, 2% *w*/*v* Peptone, 2% *w*/*v* Dextrose) liquid cultures, with 0 mg·L^−1^, 0.25 mg·L^−1^ nystatin and 0.25 mg·L^−1^ nystatin supplemented with 100 mg·L^−1^ CdS QDs, as reported in Pasquali et al. (2017) [20]. Sub-inhibitory concentrations (0.25 mg·L^−1^) of nystatin (that binds the ergosterol in the fungal cell membrane, therefore inducing the formation of pores, as reported in Bilinski et al. (1990) [29]) were added to encourage the uptake of CdS QDs into the yeast cells by increasing the membrane permeability and reducing the required concentration of QDs. To prevent aggregation, the CdS QD suspension has been thoroughly sonicated. The stability of the CdS QDs was not affected by the growth medium [16]. After 24 h shaking at 30 °C, the total RNA was extracted from 10^7^ cells·mL^−1^ with Qiagen RNeasy Mini Kit (Qiagen, Velno, The Netherlands). Five replicates for each RNA extraction were performed. The genome-wide transcriptome was acquired using the GeneChip Yeast Genome 2.0 Array (Affymetrix, Santa Clara, CA, USA). For each treatment condition, two aliquots of total RNA extract (500 ng) were amplified, biotin-labeled, hybridized to the microarray, and the outputs were analyzed according to the protocols provided by Biolitix AG (Witterswill, Switzerland). The data have been deposited in the NCBI GEO database (accession number GSE125759). Additional methods related to the validation of microarray data are reported in the Supplementary Materials.

### 2.4. Determination of Glutathione Redox State and Mitochondrial Morphotype

The glutathione redox state has been tested on 10^5^ cells·mL ^−1^ grown in SC (0.67% *w*/*v* yeast nitrogen base with aminoacids, 2% *w*/*v* dextrose; Sigma-Aldrich, St. Louis, MO, USA) medium, treated for 24 h with 50 mg·L^−1^ of CdS QDs; the cellular extract and glutathione levels were determined as described in Pasquali et al. (2017) [20]. To determine the mitochondrial morphotype wild type strain, tolerant and sensitive mutants were transformed with the plasmid pYX-142 mtRFP as described by Pasquali et al. (2017) [20]. Each strain was grown in the selective SC-LEU medium (0.67% *w*/*v* yeast nitrogen base w/o aminoacids, 0.16% *w*/*v* yeast synthetic drop-out medium supplement w/o leucine, 2% *w*/*v* dextrose; Sigma-Aldrich, St. Louis, MO, USA) containing 50 mg·L^−1^ of CdS QDs, for a 24 h treatment. Microscope images were taken using an Axio Imager 2 (Zeiss, Oberkochen, Germany). The experiments were performed in triplicate. Additional methods related to the growth characteristics data of the wild type strain, and the tolerant and sensitive mutants are reported in the Supplementary Materials.

### 2.5. Statistical Analysis

Raw microarray data were analyzed using the Affymetrix Expression Console v1.4.0 (www.affymetrix.com/analysis/, Affymetrix, Santa Clara, CA, USA). A gene expression model was obtained from normalized CEL intensities based on a perfect match only model (RMA), as described in Marmiroli et al. (2014) [26]. To evaluate the differential gene expression between each treatment, the yeast cells grown in YPD without treatment were used as a calibrator (*P* < 0.01). Heatmaps and gene clustering concerning the different conditions of treatments and the comparison between phenomic data [16] were performed with the R software (http://www.r-project.org). Differentially expressed gene classes involved in response to CdS QDs were analyzed using Gene Ontology (GO, http://www.geneontology.org/), the Kyoto Encyclopedia of Genes and Genomes (KEGG, https://www.genome.jp/kegg/), and the DAVID Bioinformatic Database v6.7 [30]. Venn’s diagrams were generated with the Venny bioinformatic tool (bioinfogp.cnb.csic.es/tools/venny/). A network analysis, reported in the Supplementary Materials, was performed using the GeneMANIA data service [31]. In order to decrease the complexity of the heatmaps and network analysis, a higher threshold (+2;−2) has been chosen.

## 3. Results

### 3.1. Microarray Data Analysis

**Figure 1:**
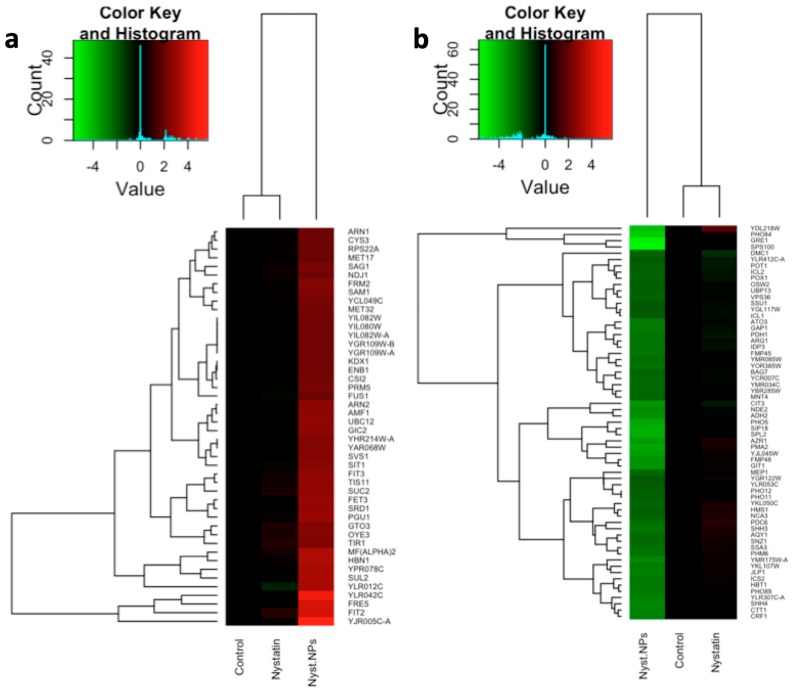
Heatmaps of the (**a**) up-regulated and (**b**) down-regulated genes highlighted from the microarray data analyses (fixed thresholds of +2;−2). Control untreated, Nystatin (0.25 mg·L^−1^) and Nystatin (0.25 mg·L^−1^) supplemented with CdS QDs (100 mg·L^−1^).

Through the microarray experiments, 312 genes up-regulated and 310 genes down-regulated in the presence of 0.25 mg·L^−1^ nystatin plus 100 mg·L^−1^ CdS QDs were identified at the fixed thresholds of +1;−1 (Tables S1 and S2), as reported in the overlapped scatterplot representing the distribution of the data of treatments vs. control untreated (Figure S1). The genes whose expression exceeds the fixed thresholds of +2;−2 were shown in the up- and down-regulated heatmaps (Figure 1).

**Figure 2:**
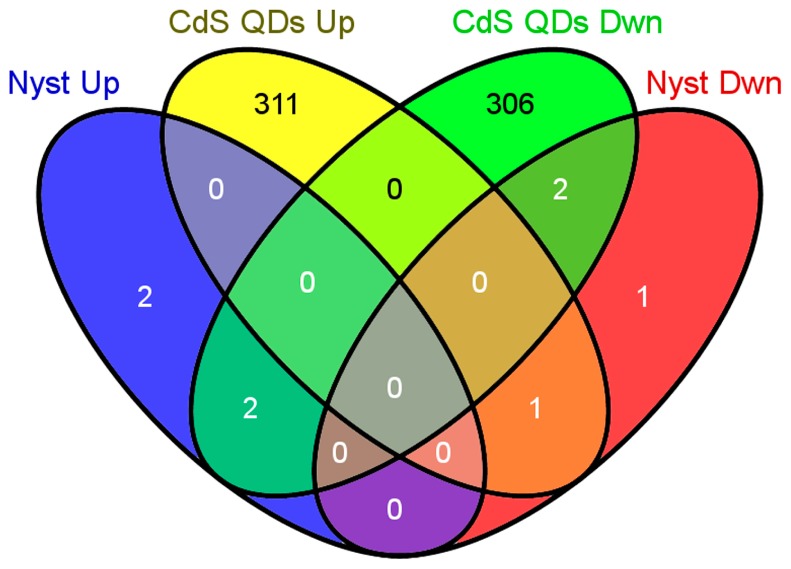
Venn’s diagram representing the overlap between up- and down-regulated genes (fixed thresholds of +1;−1) with nystatin alone and with nystatin supplemented with CdS QDs. The comparison of the four classes highlights a scant overlap between the two conditions of treatment.

A total of four genes were up-regulated and four down-regulated in the presence of 0.25 mg·L^−1^ of nystatin, and they exceeded the significance threshold of +1;−1; five out of the eight genes resulted in common with the treatment with 0.25 mg·L^−1^ nystatin plus 100 mg·L^−1^ CdS QDs, as shown by the Venn’s diagram in Figure 2.

A set of 34 genes has been used to validate through Real Time qPCR the results obtained from microarray data analysis. qPCR analyses substantially confirmed the up- or down-regulation of the genes taken into account, as reported in Figure S2.

### 3.2. Gene Ontology

**Figure 3:**
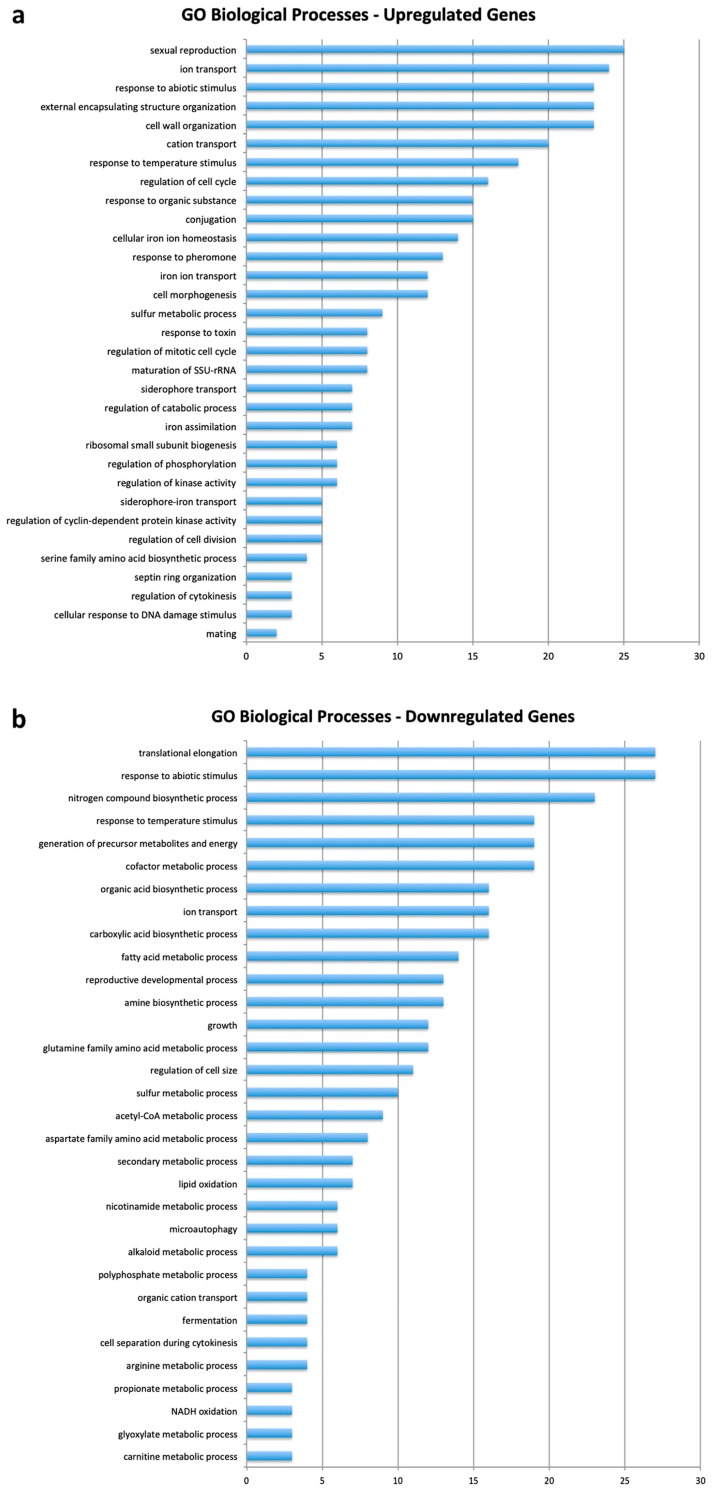
GO biological processes of (**a**) up- and (**b**) down-regulated genes derived from the microarray analysis (fixed thresholds of +1;−1).

DAVID Bioinformatic Database analyses of the genes differentially expressed in the presence or absence of QDs were enlisted with respect to the biological processes, (Figure 3, Table S3) molecular functions and cellular components (Tables S4 and S5). The main categories of up regulated genes (Figure 3a) were involved in “sexual reproduction” (10%), “ion transport” (8.7%), “cell wall organization” (8.3%), “response to abiotic stress” (8.3%), “homeostasis” (5%), and “regulation of cell cycle” (5.8%). An analysis of the down-regulated genes (Figure 3b) highlighted those categories involved in the CdS QDs’ response: “translation” (8.9%), “response to abiotic stimulus” (8.9%), “nitrogen compound processes” (7.6%), “metabolic functions” (6.3%) and “ion transport” (5.3%). The KEGG analysis of up- and down-regulated genes (Table S6) showed the metabolic pathways involved in the CdS QDs’ response: up-regulated genes were mainly involved in “ribosome assembling” (11.2%), “cell cycle” (4.7%), and “MAPK signaling” (2.9%), whereas down-regulated genes were involved in several metabolic pathways such as “amino acid biosynthesis” (2.6%), “TCA cycle” (2%) and “nitrogen metabolism” (1.7%).

The GeneMANIA data service allowed for the determination of the interaction networks for up- and down-regulated genes (Figures S3 and S4), according to the co-expression, co-localization, physical and genetic interaction data derived from literature. This also allowed the identification of sub-networks corresponding to some of the functional classes identified by prior analyses.

Among the up-regulated genes (Figure S3), the most representative functional clusters include: iron assimilation (*ARN1*, *ARN2*, *SIT1*, members of the ARN family of transporters that specifically recognize siderophore-iron chelates, *FIT2*, *FIT3*, mannoproteins of the cell wall involved in the retention of iron and *FET3*) [32,33], conjugation (*FUS1*, a membrane protein required for cell fusion, *MF(ALPHA)2*, a mating pheromone alpha-factor and *SAG1*, an alpha-agglutinin of alpha-cells) and sulphur aminoacid biosynthesis (*CYS3*, a cystathionine gamma-lyase, *MET17*, an O-acetyl homoserine-O-acetyl serine sulfhydrylase, *MET32*, a zinc-finger transcription factor involved in methionine biosynthesis and *SAM1*, a *S*-adenosylmethionine synthetase). The most connected nodes of this network are *SIT1*, which encodes a ferrioxiamine B transporter, and *ARN2*, encoding for a transporter recognizing siderophore-iron chelates.

The down-regulated genes network (Figure S4) includes genes involved in fatty acid metabolism (*CIT3*, a mitochondrial citrate and methylcitrate synthase, *ICL2*, a 2-methylisocitrate lyase of the mitochondrial matrix, *IDP3*, a peroxisomal NADP-dependent isocitrate dehydrogenase, *PDH1*, a putative 2-methylcitrate dehydratase, *POT1*, a 3-ketoacyl-CoA thiolase and *POX1*, a fatty-acyl coenzyme A oxidase), inorganic ion transport (*ATO3*, a putative ammonium transporter of the plasma membrane, *MEP1*, an ammonium specific permease, *PHO84*, *PHO89*, two inorganic phosphate transporters and *SHU1*, a component of the SHU complex that promotes DNA error-free repair) and pyridinic compound metabolism (*ADH2*, a glucose-repressible alcohol dehydrogenase II, *IDP3*, a peroxisomal NADP-dependent isocitrate dehydrogenase, *NDE2*, a mitochondrial external NADH dehydrogenase and *SNZ1*, a protein involved in vitamin B6 biosynthesis). The nodes that resulted with the highest connectivity in this network were *GRE1*, which encodes a stress-induced hydrophilin essential in the desiccation-rehydration process, and its paralog *SIP18*, which arose from the whole genome duplication [34].

### 3.3. Comparison of Two Yeast Inhibitors: Nystatin vs. CdS QDs

Nystatin, a polyene macrolide antibiotic, inhibits the formation of the yeast cell wall [29,35]. Here, the effects of the sub-inhibitory concentration of nystatin were compared either alone or with the supplement of CdS QDs. The data obtained in the case of the treatment on YPD supplemented with 0.25 mg·L^−1^ of nystatin showed that 8 genes exceeded the fixed threshold (+1;−1). Four genes showed up-regulation: *YDR366C* and *YDL218W*, which encode putative proteins with an unknown function; the transcription of *YDL218W* is induced by different kinds of stress; *IMD2* and its non-functional paralog *IMD1*, arising from a segmental duplication, which encode an inosine monophosphate protein (IMP) dehydrogenase involved in GTP biosynthesis and repressed by nutrient limitation. Four of the resulting genes were down-regulated: *DMC1*, which encodes a meiosis-specific recombinase required for double-strand break repair [36]; *MIG2*, which encodes a zinc finger transcriptional repressor involved in glucose-induced gene repression and relocated into the mitochondrion under low glucose conditions; *HXT1* which encodes a low-affinity glucose transporter; and *YOL014W,* which encodes a protein with an unknown function.

A comparison with the data obtained in the case of the treatment on YPD supplemented with 0.25 mg·L^−1^ of nystatin and YPD supplemented with 0.25 mg·L^−1^ of nystatin plus 100 mg·L^−1^ of CdS QDs showed an overlap of 5 genes (Figure 1 and Figure 2). Not surprisingly, four of them were down-regulated in the case of the treatment with nystatin plus CdS QDs: *YOL014W* and *DMC1*, already down-regulated in the case of the treatment with nystatin alone; *YDR366C* and *YDL218W*, which were instead up-regulated in the presence of nystatin only. The only gene showing a significant up-regulation in the case of the treatment with nystatin plus CdS QDs was *MIG2* [37], which was down-regulated under the treatment with 0.25 mg·L^−1^ of nystatin. Therefore, CdS QDs exert an inhibitory effect largely different from the antifungal nystatin.

### 3.4. Comparison with Cadmium Ion Response

A comparison with studies related to up- and down-regulated genes involved in the Cd^2+^ response, identified by Jin et al. (2008) [38], showed that only eight genes were commonly modulated during the CdS QDs’ response (Figure S5). Among the up-regulated genes were: *CYS3*, involved in the metabolism of cysteine; *SUL2*, which encodes a sulfate permease [39], *FET3* and *FRE5* (paralog of Fre2p), both encoding proteins involved in the oxidation of ionic iron. The down-regulated genes were: *PHO5*, phosphatase induced by phosphate starvation; *IDP3*, which codifies for a peroxisomal NADP-dependent isocitrate dehydrogenase; *ARG1*, involved in the arginine biosynthetic pathway and *YBR285W*, whose gene product function is unknown. Similar conclusions were reported in studies on *Arabidopsis thaliana* and human cells [21,26].

### 3.5. Comparison between Phenomics and Transcriptomics Data

**Figure 4:**
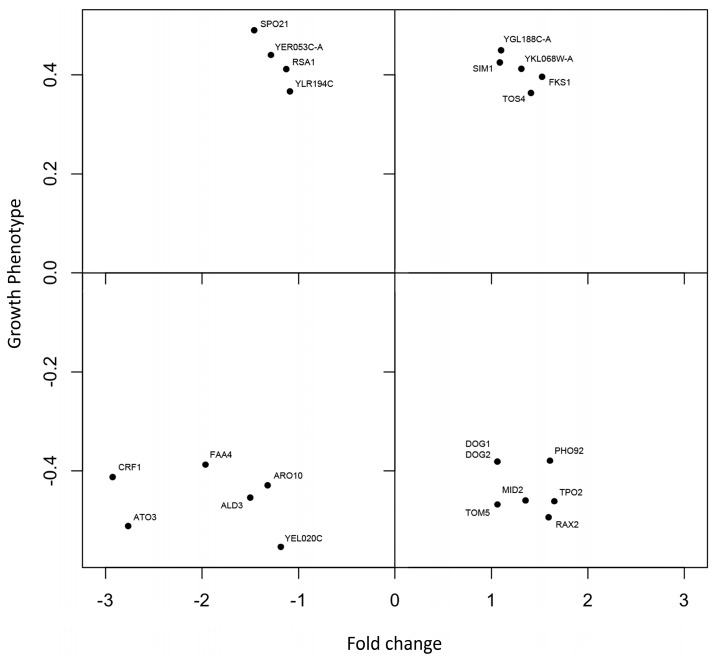
Scatter plot representing the comparison between the fold change (increase/decrease) in gene expression, from microarray analyses, and the growth phenotype (GP) knock-out mutant screening, expressed as tolerant and sensitive phenotypes [16].

A set of 226 genes (respectively, 112, which were deleted, induced sensitivity, and 114 induced tolerance to CdS QDs) reported in Marmiroli et al. (2016) [16] was compared with the data obtained from the microarray experiments to highlight key genes involved in the response to CdS QDs (Figure 4, Table 1). Among those that were both up-regulated and whose deletion was leading to a tolerant phenotype, the following were observed: the genes *SIM1*, that encodes for the SUN family protein (Sim1p, Uth1p, Nca3p, Sun4p) implicated in DNA replication, and mitochondrial biogenesis [40]; *TOS4*, a putative transcription factor, involved in the DNA replication checkpoint response, whose relative distribution into the nucleus increases upon DNA replication stress; *FKS1*, that encodes for the catalytic subunit of 1,3-beta-D-glucan synthase; and *YGL188C-A*, *YKL068W-A,* whose functions are unknown.

Among the genes that resulted significantly in down-regulation thorough the microarray analysis, but whose deletion lead to a tolerant phenotype, we observed the genes: *YER053C-A*, that encodes for a gene product with an unknown function, but whose abundance increases in response to DNA replication stress; *YLR194C*, a structural constituent of the cell wall, whose expression is up-regulated in response to cell wall stress; *SPO21*, which encodes for a component of the meiotic outer plaque of the spindle pole body; and *RSA1*, which is involved in the 60S ribosomal subunits assembly. On the other hand, the genes that resulted in up-regulation and whose deletion lead to a sensitive phenotype were: *PHO92*, a post-transcriptional regulator of the phosphate metabolism; regulating Pho4 mRNA stability by binding to Pho4’s 3’UTR in a phosphate-dependent manner; *TPO2*, that encodes for a polyamine transporter of the major facilitator superfamily protein; *DOG1*, *DOG2*, which are respectively members of a low molecular weight phosphatases family, induced by oxidative and osmotic stress; *MID2*, which encodes for a O-glycosylated plasma membrane protein that acts as a sensor for cell wall integrity signaling; *TOM5*, a component of the TOM (Translocase of Outer Membrane) complex; responsible for the recognition and import of all mitochondrial directed proteins [20], and *RAX2*, a N-glycosylated protein involved in budding. Among those genes that showed both down-regulation and whose deletion resulted in a sensitive phenotype were observed: *CRF1*, a transcriptional co-repressor involved in the repression of ribosomal protein gene transcription; *ARO10*, which encodes for a phenylpyruvate decarboxylase involved in protein N-terminal methionine and alanine catabolism; *ATO3*, a plasma membrane protein, putatively involved in the ammonia export from the cell; *ALD3*, a cytoplasmic aldehyde dehydrogenase, involved in beta-alanine synthesis, whose expression is induced by stress and repressed by glucose [16]; *FAA4*, a long chain fatty acyl-CoA synthetase; and *YEL020C*, whose gene product has an unknown function.

### 3.6. Confirmation of Growth Characteristics in a Pool of Selected Mutants

The entire set of haploid barcoded deletion mutants described by Marmiroli et al. (2016) and Pasquali et al. (2017) [16,20] were confirmed for their growth characteristics, and a pool of selected tolerant and sensitive mutants with particularly interesting features were chosen for further characterization (Tables S7 and S8). The growth phenotype was also monitored with carbon sources other than glucose, in YP and SC media supplemented with CdS QDs (Tables S7 and S8). The treatment with CdS QDs in SC occurred in the absence of nystatin and the results on the sensitivity/tolerance of the selected pool of mutants were confirmed (Tables S7 and S8).

### 3.7. Physiological and Molecular Tests on a Pool of Selected Tolerant and Sensitive Mutants

**Figure 5:**
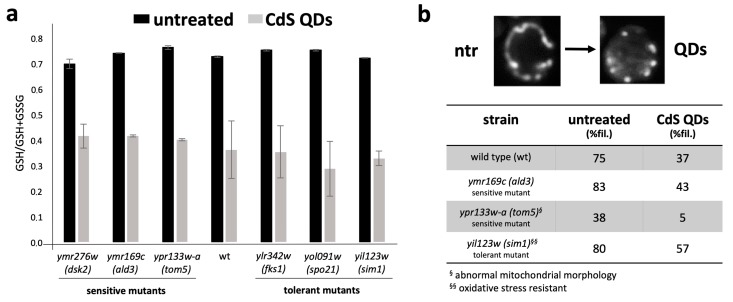
(**a**) Histogram reporting the GSH/GSH+GSSG ratio in wild type yeast cells and in tolerant or sensitive mutants. Untreated (black, ntr), treated (grey, QDs) with 50 mg·L^−1^ of CdS QDs. (**b**) Top: pictures depicting the percent of filamentous mitochondria in untreated and treated (50 mg·L^−1^) cells, respectively. The cells were transformed with a plasmid expressing mitochondrial RFP [20]. Bottom: table indicating the percentage of filamentous mitochondria in untreated (ntr) and treated (QDs) yeast samples.

Three sensitive mutants and three tolerant mutants were considered on the basis of the expression of the corresponding deleted gene in the wild type condition in the presence of CdS QDs. In these mutants, the glutathione state was compared in the wild type and mutants, tolerant or sensitive to CdS QDs. The data reported in Figure 5, Tables S7 and S8, showed a similar decrease in the GSH/GSH+GSSG ratio in all of the treated samples in both the tolerant and sensitive mutants. This trend indicated a general increase in the formation of the glutathione oxidized form. This increase however was smaller in the tolerant mutants, especially for *spo21* (Figure 5a). In parallel, mutants were transformed with a plasmid expressing a Red Fluorescent Protein (RFP), which localizes in the mitochondria. This allowed in vivo a direct observation of the organelles morphology. The results obtained revealed that the percentage of filamentous mitochondria (normal and functional) [20] diminished when cells were treated with CdS QDs, but in oxidative stress resistant mutants (e.g., *sim1*) this reduction was smaller (Figure 5b). Of interest was the behaviour of *tom5*, a sensitive mutant, that without CdS QDs had a very low amount of morphologically normal and functional mitochondria, that almost disappeared when exposed to CdS QDs (Figure 5b). Nitric oxide (NO), another indicator of cell stress, did not change after the treatment in either the tolerant mutants or the wild type (Table S8).

## 4. Discussion

### 4.1. Sub-Inhibitory Concentration of Nystatin Did Not Influence Yeast Response to CdS QDs

The growth phenotype (GP) and the growth characteristics of the wild type and selected mutants did not change in the presence or absence of nystatin (YPD + Nyst and SC), in complete (YP) media with fermentable (dextrose and galactose) or oxidizable (glycerol) carbon sources (Tables S7 and S8). Moreover, the utilization of nystatin did not significantly perturb the differentially expressed genes. After the treatment with nystatin only, 8 genes were up or down regulated above or below the fixed threshold (+1;−1); their functions were related to the general stress response, transcription, and glucose metabolism; three genes were encoded for proteins of unknown functions. From a physiological point of view, nystatin and CdS QDs are both inhibitors of yeast growth and proliferation. Their action is synergic and can be modulated by the ratio between the amount of nystatin and the one of CdS QDs. This may open some perspective in the search for new agents, or combinations, to stop yeast growth in pathogenic conditions.

### 4.2. Cadmium Ion Response Comparison

An analysis of the data of the gene expression underlined the small overlap (1.3%; 8 out of 622 genes) between the Cd^2+^ and CdS QDs’ response. These results confirm what was observed in yeast in *Arabidopsis thaliana* and in human cells [16,21,26]. This finding, with the observation that in the growth medium CdS QDs are highly stable, indicates that the pattern of the response depends specifically on the nanomaterial [16,20].

Moreover, these results pointed to the fact that the transcriptional response to some ENMs and the one to the corresponding ion can be very different, a result reported also when other ENMs were studied [41,42].

### 4.3. Biological Function of the Genes Involved in the Response of Yeast to CdS QDs

The main GO biological processes enriched from the microarray analyses were: ion transport, response to general stress, cell wall organization, metabolic functions, and regulation of the cell cycle. These processes constituted the “core response” to CdS QDs, and were also observed in different model organisms from simple to higher eukaryotes, as well as in human cells [16,21,26]. The preservation of the main response functions, even though not accompanied by the conservation of specific genes across the different species (especially when considering phylogenetically distant organisms), highlighted the importance of yeast as a fundamental biological resource for in vivo-in vitro nanotoxicology studies [20].

From a physiological and molecular point of view, it has been demonstrated that ENMs increase the Reactive Oxygen Species (ROS) production within the cells. These excessive ROS interact negatively with all cell compartments, in particular affecting cell membranes and the energy production at the level of the mitochondria [43]. However, it remains unclear whether the generation of intracellular ROS could derive only from a direct interaction at the level of the mitochondria or if the ROS production at the level of the cytosol was also involved [43]. Measures of the cell redox state utilizing parameters related to the GSH oxidation state confirmed that in both tolerant and sensitive mutants, treated with CdS QDs, the oxidation level of glutathione was higher than in the not treated control (Figure 5, Tables S7 and S8). The oxidative stress response, in yeast, can be considered a biomarker of the exposure to CdS QDs, whose behaviour is dependent on the ENM physico-chemical characteristics (e.g., particle size, surface charge, composition, stability, aggregation, and presence of coating), on the conditions of the treatment and, more importantly, on the organism [28,43]. Considering these parameters, the literature reports several examples in which, for instance, Au ENMs was able to stimulate oxidative stress [23], whereas Ag or ZnO ENMs’ response was independent from a direct oxidative response pathway, but dependent mainly on the ENMs’ stability [44,45].

Other relevant processes found to be strictly related to the core response were the protein synthesis, TCA cycle, and nitrogen metabolism. In particular, as reported by the recent literature [20,43], those processes might be associated to the mitochondrial functionality, confirming that mitochondria are a preferential target of ENMs [22,46] and specifically of CdS QDs [16,21]. There is strong experimental evidence that the CdS QDs-induced ROS and oxidative stress lead directly to a mitochondrial-mediated cell apoptosis. As described by Pasquali et al. (2017) [20], CdS QDs hinder the mitochondrial functionality, triggering a cytochrome-c dependent apoptotic mechanism, observed also in HepG2 human cells [21]. Moreover, the regulation of the sexual reproduction seems to be considerably involved in the CdS QDs’ response and related closely to nitrogen starvation [47].

Observing in vivo fluorescently labelled mitochondria, it was clear how these structures were impaired by CdS QDs: upon treatment, their structure becomes increasingly punctuated instead of filamentous (Figure 5, Tables S7 and S8). Indeed, each strain could display a different basal level of mitochondria filaments, which was minimal in the *tom5* sensitive mutant, which has a low level of mitochondrial function. The amount of filamentous mitochondria upon treatment with CdS QDs seems to decrease more in the sensitive than in the tolerant mutants (Figure 5b, Tables S7 and S8).

### 4.4. Comparison between Phenomic and Transcriptomic Data

The genes obtained from the array analysis were compared with those associated with the growth screening [16] (Figure 4). The two groups of genes that were up- and down-regulated showed different types of correlation between the level of the gene expression in the microarray and the growth phenotype associated with some genes in the knock-out mutants (Figure 4, Table 1). Each of these groups is composed of two sets: in one the level of the expression of the gene is coherent with the observed growth phenotype (both a tolerant phenotype and an up-regulated gene, or a sensitive phenotype and a down-regulated gene), in the other group there is an apparent separation between the level of gene expression and the observed growth phenotype (a tolerant phenotype and a down-regulated gene or a sensitive phenotype with an up-regulated gene). The last two categories in which the two screenings gave results, which were clearly in “repulsion”, are particularly interesting.

Regarding those genes that were up-regulated, but whose deletion produced a tolerant phenotype (Table 1, + +), excluding *YGL188C-A*, *YKL068W-A* with a still unknown function, *SIM1*, *TOS4*, and *FKS1* were putatively involved in common processes related to the regulation of cellular duplication at different levels (from DNA to the cell wall) [48]. That might be explained by the fact that the CdS QDs’ stress can stimulate different types of responses, including cell division. The tolerant phenotypes of the knock-out mutants compared with the wild type strain are more difficult to explain. Tentatively, it might be related to the gene duplication of *SIM1*, *TOS4* and *FKS1*, which makes their functions apparently not essential. However, as reported by Pasquali et al. (2017) [20], *FKS1* has been found to have a key role in the mitochondrial integrity and functionality under CdS QDs’ response. Furthermore, the deletion of *sim1* resulted in oxidative stress resistance (Figure 5), which might suggest how the response to ROS is predominant in CdS QD exposure [16,43].

In the case of down-regulated genes, whose deletion produced tolerant phenotypes as compared to the wild type strain (*YER053C-A*, *YLR194C*, *SPO21*, *RSA1*)(Table 1, + −), the functions and processes in which their gene products are involved were similar to those observed for the set of up-regulated genes and tolerant phenotypes: stress related-DNA duplication and cell wall integrity. Interestingly, the effects on the expression of this set of genes seemed to be contrary to what was expected during the stress conditions, according to their functions. This might suggest that the presence of possible pathways is able to complement the role of these genes in the wild type strain, as confirmed by the knock-out mutants, in which tolerant phenotypes were observed.

The up-regulated genes in the wild type strain, but whose deletion produced sensitive phenotypes, were: *PHO92*, *TPO2*, *DOG1*, *DOG2*, *MID2*, *TOM5*, and *RAX2* (Figure 4, Table 1, − +). Concerning the Dog1 and Dog2 gene products, which are involved in the general response to osmotic and oxidative stress, they maintained the general trend of the oxidative stress response shown during the CdS QD exposure. Mid2 protein is a fundamental component of the cell wall that acts as a sensor of abiotic stress, specifically in relation to the integrity at the level of the cellular membrane, whose importance in CdS QDs’ response has already been mentioned above. *PHO92* and *TPO2* are implicated respectively in the regulation of the response to phosphate starvation and polyamine export, across different organisms, whose functions are considered one of the main indicators of ENMs’ stress [49,50]. Rax2 protein is implicated, in budding processes that, as in the case of Spo21, have been highlighted in CdS QDs’ exposure. *TOM5* is a major regulator of mitochondrial protein trafficking, evidencing consistently the importance of the mitochondrion in the response to CdS QDs and to ENMs, in general. Indeed, its absence causes different mitochondria morphologies characterized by the presence of fewer filaments that almost disappear upon treatment with CdS QDs (Figure 5).

The genes that are down-regulated in the wild type strain, and whose deletion produced sensitive phenotypes (Table 1, − −), were: *CRF1*, *ARO10*, *ATO3*, *ALD3*, *FAA4*, and *YEL020C*, the latter having an unknown function. Aro10, Faa4 and Ald3 proteins showed cytoplasmic key roles in metabolic activities strongly regulated by abiotic stress. The Ato3 protein is implicated, as in the case of the Tpo2 protein, in the nitrogen compounds export. Crf1 is a co-repressor that has been found to be involved in the regulation of ribosomal proteins expression. Its down-regulation suggested a promotion of protein synthesis during CdS QDs’ response; however, Crf1 has been found to be a strain specific regulator [51], which makes it more complex to fit this gene into a possible systemic mechanism of response to CdS QDs.

## 5. Conclusions

CdS QDs’ response, and more generally the response to ENMs, has been thoroughly investigated across different species, from bacteria to human [42,43,52,53]. The results obtained clearly showed how CdS QDs triggered unique response mechanisms (e.g., the response to abiotic stimuli and cellular compartment organization), when acompared with those related to the Cd^2+^ ion alone. This underlines the importance of distinguishing between the effects given by the different forms of metal-based compounds (bulk, ion, nano) [16,28]. This point becomes fundamental, when also considering the QDs’ physico-chemical properties (size, surface charge, composition, stability, aggregation) related to the nano form compared to the bulk and ion forms, along with the possible scenarios of environmental and human exposure [5,19,54,55].

Yeast, as a single cell eukaryotic organism, has been used for multidisciplinary approaches involving mutant growth phenotypes screening and high-throughput genomic, transcriptomic, and proteomic analyses. Considering the preservation of cellular (and mitochondrial) functions with higher eukaryotes and humans [16,20,21], yeast can be regarded as a versatile and robust biological tool for targeted toxicological strategies. Furthermore, many human diseases can be associated with dysfunctions at the cellular or mitochondrial level, such as cancer, diabetes, and cardiovascular or neurodegenerative diseases [24,56,57]. The comparison of different methodologies, from genomic to phenotypic screening, opens up the possibility of a more thoughtful comparison between in vivo and in vitro results.

## Figures and Tables

**Table 1:** Genes selected after the comparison of the phenotype screening and microarray analysis, represented in Figure 4. (+ +) genes that showed a tolerant knock-out phenotype and were up-regulated in the wild type strain; (+ −) genes that showed a tolerant knock-out phenotype and were down-regulated in the wild type strain; (− +) genes that showed a sensitive knock-out phenotype and were up-regulated in the wild type strain; (− −) genes that showed a sensitive knock-out phenotype and were down-regulated in the wild type strain.

Gene ID	Function
**+ +**	
*FKS1*	Catalytic subunit of 1,3-beta-D-glucan synthase; functionally redundant with alternate catalytic subunit Gsc2p; binds to regulatory subunit Rho1p; involved in cell wall synthesis and maintenance; localizes to sites of cell wall remodeling; FKS1 has a paralog, GSC2, that arose from the whole genome duplication
*SIM1*	Protein of the SUN family (Sim1p, Uth1p, Nca3p, Sun4p); may participate in DNA replication; promoter contains SCB regulation box at −300 bp indicating that expression may be cell cycle-regulated; SIM1 has a paralog, SUN4, that arose from the whole genome duplication
*TOS4*	Putative transcription factor, contains Forkhead Associated domain; found associated with chromatin; target of SBF transcription factor; expression is periodic and peaks in G1; involved in DNA replication checkpoint response; interacts with Rpd3 and Set3 histone deacetylase (HDAC) complexes; APCC(Cdh1) substrate; relative distribution to the nucleus increases upon DNA replication stress; TOS4 has a paralog, PLM2, that arose from the whole genome duplication
*YGL188C-A*	Unknown function
*YKL068W-A*	Unknown function
**+ −**	
*RSA1*	Protein involved in the assembly of 60S ribosomal subunits; functionally interacts with Dbp6p; functions in a late nucleoplasmic step of the assembly
*SPO21*	Component of the meiotic outer plaque of the spindle pole body; involved in modifying the meiotic outer plaque that is required prior to the prospore membrane formation; SPO21 has a paralog, YSW1, that arose from the whole genome duplication
*YER053C-A*	Unknown function; green fluorescent protein (GFP)-fusion protein localizes to the endoplasmic reticulum; protein abundance increases in response to DNA replication stress
*YLR194C*	Structural constituent of the cell wall; attached to the plasma membrane by a GPI-anchor; expression is up-regulated in response to cell wall stress
**− +**	
*DOG1*	2-deoxyglucose-6-phosphate phosphatase; member of a family of low molecular weight phosphatases; confers 2-deoxyglucose resistance when overexpressed, in vivo substrate has not yet been identified; DOG1 has a paralog, DOG2, that arose from a single-locus duplication
*DOG2*	2-deoxyglucose-6-phosphate phosphatase; member of a family of low molecular weight phosphatases, induced by oxidative and osmotic stress, confers 2-deoxyglucose resistance when overexpressed; DOG2 has a paralog, DOG1, that arose from a single-locus duplication
*MID2*	O-glycosylated plasma membrane protein; acts as a sensor for cell wall integrity signaling and activates the pathway; interacts with Rom2p, a guanine nucleotide exchange factor for Rho1p, and with the cell integrity pathway protein Zeo1p; MID2 has a paralog, MTL1, that arose from the whole genome duplication
*PHO92*	Posttranscriptional regulator of phosphate metabolism; facilitates PHO4 mRNA degradation by interacting with Pop2p; regulates PHO4 mRNA stability by binding to PHO4’s 3’UTR in a phosphate-dependent manner; contains highly conserved YTH (YT521-B Homology) domain that exhibits RNA-binding activity; functional homolog of human YTHDF2
*RAX2*	N-glycosylated protein; involved in the maintenance of bud site selection during bipolar budding; localization requires Rax1p; RAX2 mRNA stability is regulated by Mpt5p
*TOM5*	Component of the TOM (translocase of outer membrane) complex; responsible for the recognition and initial import of all mitochondrially directed proteins; involved in the etransfer of precursors from the Tom70p and Tom20p receptors to the Tom40p pore
*TPO2*	Polyamine transporter of the major facilitator superfamily; member of the 12-spanner drug:H(+) antiporter DHA1 family; specific for spermine; localizes to the plasma membrane; transcription of TPO2 is regulated by Haa1p; TPO2 has a paralog, TPO3, that arose from the whole genome duplication
**− −**	
*ALD3*	Cytoplasmic aldehyde dehydrogenase; involved in the beta-alanine synthesis; uses NAD+ as the preferred coenzyme; very similar to Ald2p; expression is induced by stress and repressed by glucose
*ARO10*	Phenylpyruvate decarboxylase; catalyzes decarboxylation of phenylpyruvate to phenylacetaldehyde, which is the first specific step in the Ehrlich pathway; involved in protein N-terminal Met and Ala catabolism
*ATO3*	Plasma membrane protein, putative ammonium transporter; regulation pattern suggests a possible role in the export of ammonia from the cell; phosphorylated in mitochondria; member of the TC 9.B.33 YaaH family of putative transporters
*CRF1*	Transcriptional corepressor; involved in the repression of ribosomal protein (RP) gene transcription via the TOR signaling pathway which promotes the accumulation of Crf1p in the nucleus; role in the repression of RP genes varies by strain; CRF1 has a paralog, IFH1, that arose from the whole genome duplication
*FAA4*	Long chain fatty acyl-CoA synthetase; activates imported fatty acids with a preference for C12:0-C16:0 chain lengths; functions in long chain fatty acid import; important for survival during stationary phase; localized to lipid particles; involved in the sphingolipid-to-glycerolipid metabolism; forms cytoplasmic foci upon DNA replication stress; FAA4 has a paralog, FAA1, that arose from the whole genome duplication
*YEL020C*	Protein of unknown function with low sequence identity to Pdc1p

## Supplementary Materials

The following are available online at https://www.mdpi.com/2079-4991/9/4/512/s1. Supplementary methods related to the Real Time qPCR validation of microarray data and determination growth characteristics. Figure S1. Scatter plot representing the distribution of microarray expression data. Figure S2. Heatmap representing the comparison of the panel of 34 genes utilized for microarray validation by Real Time qPCR. Figure S3. Network of interaction of up-regulated genes derived from microarray analysis. Figure S4. Network of interaction of down-regulated genes derived from microarray analysis. Figure S5. Venn’s diagram representing the overlap between up- and down-regulated genes during CdS QDs’ exposure and Cd^2+^ ions exposure. Table S1. List of up-regulated genes highlighted after the CdS QDs’ exposure in *S. cerevisiae*. Table S2. List of down-regulated genes highlighted after the CdS QDs’ exposure in *S. cerevisiae*. Table S3. Biological processes after CdS QDs’ exposure in *S. cerevisiae*. Table S4. Molecular functions after CdS QDs’ exposure in *S. cerevisiae*. Table S5. Cellular components after CdS QDs’ exposure in *S. cerevisiae*. Table S6. (KEGG) Biochemical pathways after CdS QDs’ exposure in *S. cerevisiae*. Table S7. Comparison of wild type strain versus sensitive and tolerant mutants. Table S8. Comparison of wild type strain and tolerant mutants.
