# Supplementary material for: In Vivo-In Vitro Comparative Toxicology of Cadmium Sulphide Quantum Dots in the Model Organism Saccharomyces cerevisiae
Source: Nanomaterials (Basel). 2019 Apr 2;9(4):512. doi: 10.3390/nano9040512 (PMC6523553; doi:10.3390/nano9040512)
Supplement: Supplementary file 1 [file nanomaterials-09-00512-s001.zip › supplementary-proof done/Pagano 2019 SM1 18-02-2019.pdf]

Supplementary Materials (SM1)

In Vivo-In Vitro Comparative Toxicology of Cadmium Sulphide Quantum Dots in the Model Organism *Saccharomyces cerevisiae*

Luca Pagano [1], Marina Caldara [1], Marco Villani [2], Andrea Zappettini [2], Nelson Marmiroli [1,3] and Marta Marmiroli [1,*]

[1] Department of Chemistry, Life Sciences and Environmental Sustainability, University of Parma, 43123 Parma, Italy; luca.pagano@unipr.it (L.P.); marina.caldara@unipr.it (M.C.); nelson.marmiroli@unipr.it (N.M.)
[2] IMEM-CNR, 43123 Parma, Italy; marco.villani@imem.cnr.it (M.V.); andrea.zappettini@imem.cnr.it (A.Z.)
[3] Consorzio Interuniversitario Nazionale per le Scienze Ambientali (CINSA), University of Parma, 43123 Parma, Italy
* Correspondence: marta.marmiroli@unipr.it; Tel.: +39-0521905698

List of the Supplementary Materials:

Supplementary methods related to Real Time qPCR validation of microarray data and determination growth characteristics.

Figure S1. Scatter plot representing the distribution of microarray expression data.

Figure S2. Heatmap representing the comparison of the panel of 34 genes utilized for microarray validation by Real Time qPCR.

Figure S3. Network of interaction of up-regulated genes derived from microarray analysis.

Figure S4. Network of interaction of down-regulated genes derived from microarray analysis.

Figure S5. Venn's diagram representing the overlap between up- and down-regulated genes during CdS QDs exposure and Cd^{2+} ions exposure.

Table S1. List of up-regulated genes highlighted after the CdS QDs exposure in *S. cerevisiae*.

Table S2. List of down-regulated genes highlighted after the CdS QDs exposure in *S. cerevisiae*.

Table S3. Biological processes after CdS QDs exposure in *S. cerevisiae*.

Table S4. Molecular functions after CdS QDs exposure in *S. cerevisiae*.

Table S5. Cellular components after CdS QDs exposure in *S. cerevisiae*.

Table S6. (KEGG) Biochemical pathways after CdS QDs exposure in *S. cerevisiae*.

Table S7. Comparison of wild type strain versus sensitive and tolerant mutants.

Table S8. Comparison of wild type strain and tolerant mutants.

Supplementary Tables (SM2) are reported in excel format.

Supplementary Methods

Real Time qPCR validation of modulated genes

Reverse transcription was performed on 1 μg of the yeast total RNA extracted, using the Qiagen QuantiTect Reverse Transcription Kit (Qiagen, Velno, Netherlands). Amplifications were carried out using the Applied Biosystems Power SYBR Green Master Mix (Applied Biosystems, Foster City, CA, USA) in an optical 96-well plate with the Applied Biosystems ABI PRISM 7900HT Sequence Detection System. RNA retrotranscription were performed on the same samples used in the microarray

experiments (YPD liquid medium supplemented with 0 mg L^{-1}, 0.25 mg L^{-1} nystatin and 0.25 mg L^{-1} nystatin plus 100 mg L^{-1} CdS QDs). Specific primers for each gene selected from the microarray experiments were designed, using the Applied Biosystems SDS 2.3 software and the following thermal profile: 95°C for 10′, 95°C for 15″ and 60°C for 60″ (for 40 cycles). Synthesized primers (Sigma-Aldrich, St. Louis, MO, USA), were assessed by Real Time quantitative PCR in four serial dilutions of synthesized cDNA (1, 1:10, 1:100, 1:1000). Relative expression was estimated through ΔΔCt method, using *PDA1* (pyruvate dehydrogenase, subunit α) as housekeeping gene. The relative quantity of the transcript assayed in each RNA sample was determined by normalization on the housekeeping gene expression level and calculated as an arithmetic mean of the three independent repeated reactions.

Determination of growth characteristics

Data reported as growth characteristic represent the number of duplications (N Dupl) and the time of duplication (t Dupl) of each strain calculated upon 24h of growth in the different media: YPD or SC (Synthetic Complete, 0.67% w/v yeast nitrogen base with aminoacids, 2% w/v dextrose; Sigma-Aldrich, St. Louis, MO) media, with or without the addition of nystatin (0.5 mg L^{-1}), CdS QDs (50 mg L^{-1}) or nystatin supplemented with CdS QDs (50 mg L^{-1} or 200 mg L^{-1}). The percentage of dead cells, which incorporate Propidium Iodide PI (10 mg L^{-1}), has been measured on 10^6 cells mL^{-1}, by flow cytometry (NovoCyte, ACEA Biosciences, Inc., San Diego, CA, USA), after 24h treatment on SC supplemented

with CdS QDs (50 mg L^{-1}).

The same extract used for glutathione redox state measurement has been used for NO colorimetric assay, performed with the Nitric Oxide Non-Enzymatic Assay Kit (Oxford Biomedical Research, Rochester Hills, MI, USA). Evaluation of Nitric Oxide (NO) has been performed upon growth for 4h on SC or SC supplemented with CdS QDs (50 mg L^{-1}). Experiments were performed in triplicate.

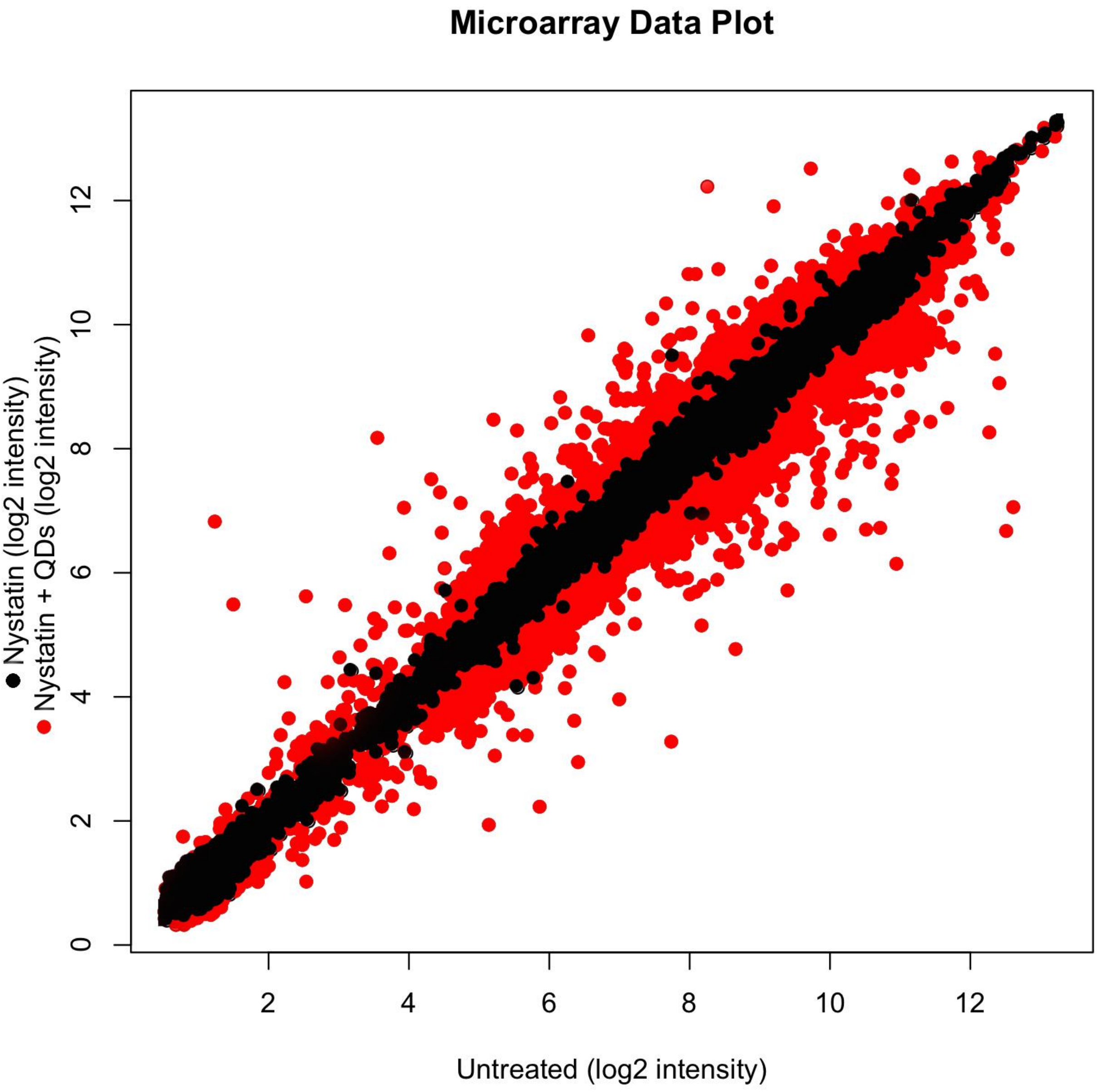

Figure S1. Scatter plot representing the distribution of expression data from nystatin (0.25 mg L^{-1}), in black, and nystatin (0.25 mg L^{-1}) supplemented with CdS QDs (100 mg L^{-1}), in red, when compared with the control untreated.

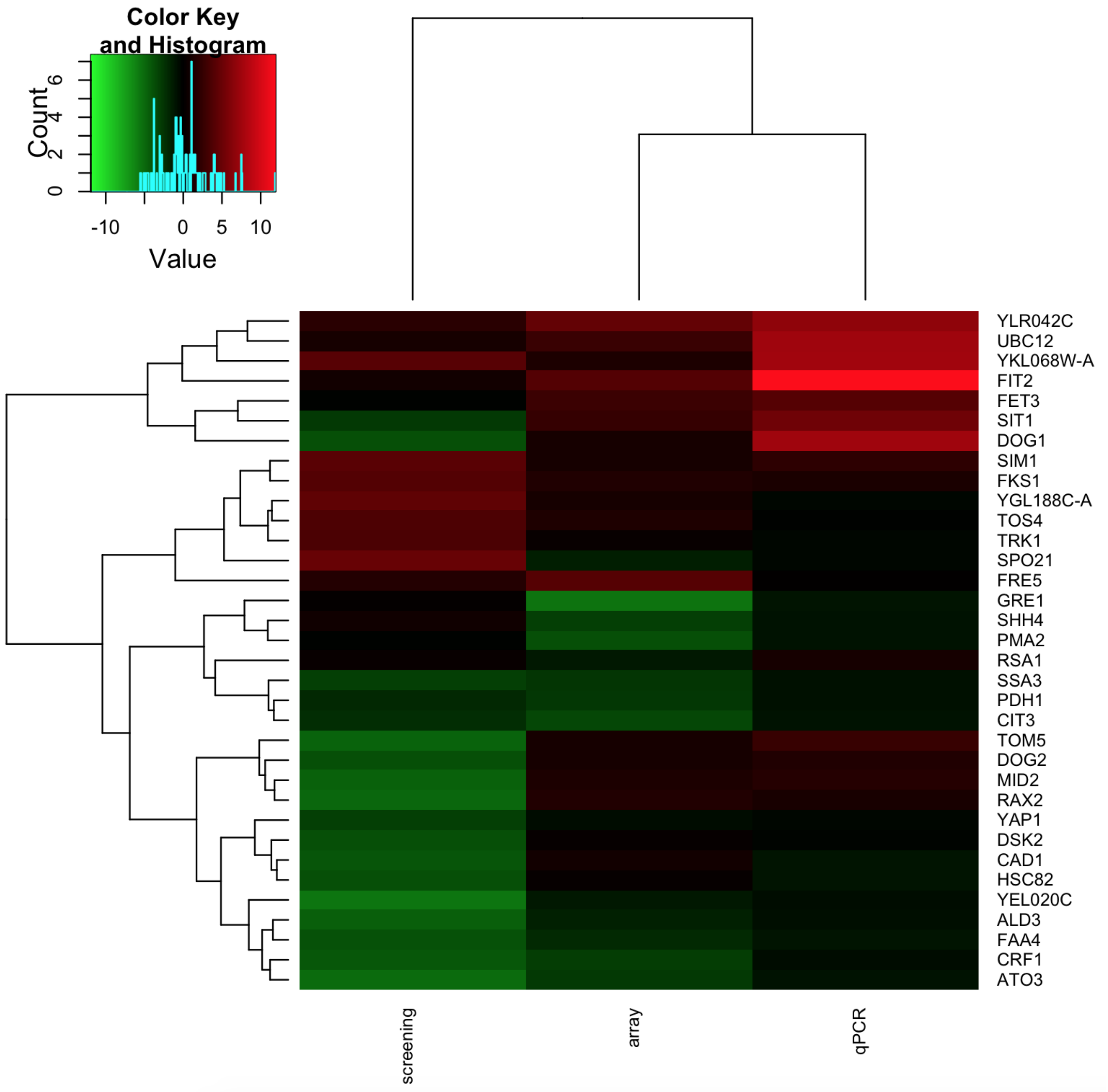

Figure S2. Heatmap representing the comparison of the panel of 34 genes utilized for microarray validation by Real Time qPCR. Data are reported from the screening of the phenotype of knock-out mutants (Marmiroli *et al.*, 2016) and the related genes expression levels in the wild type strain, both from microarray and qPCR validation.

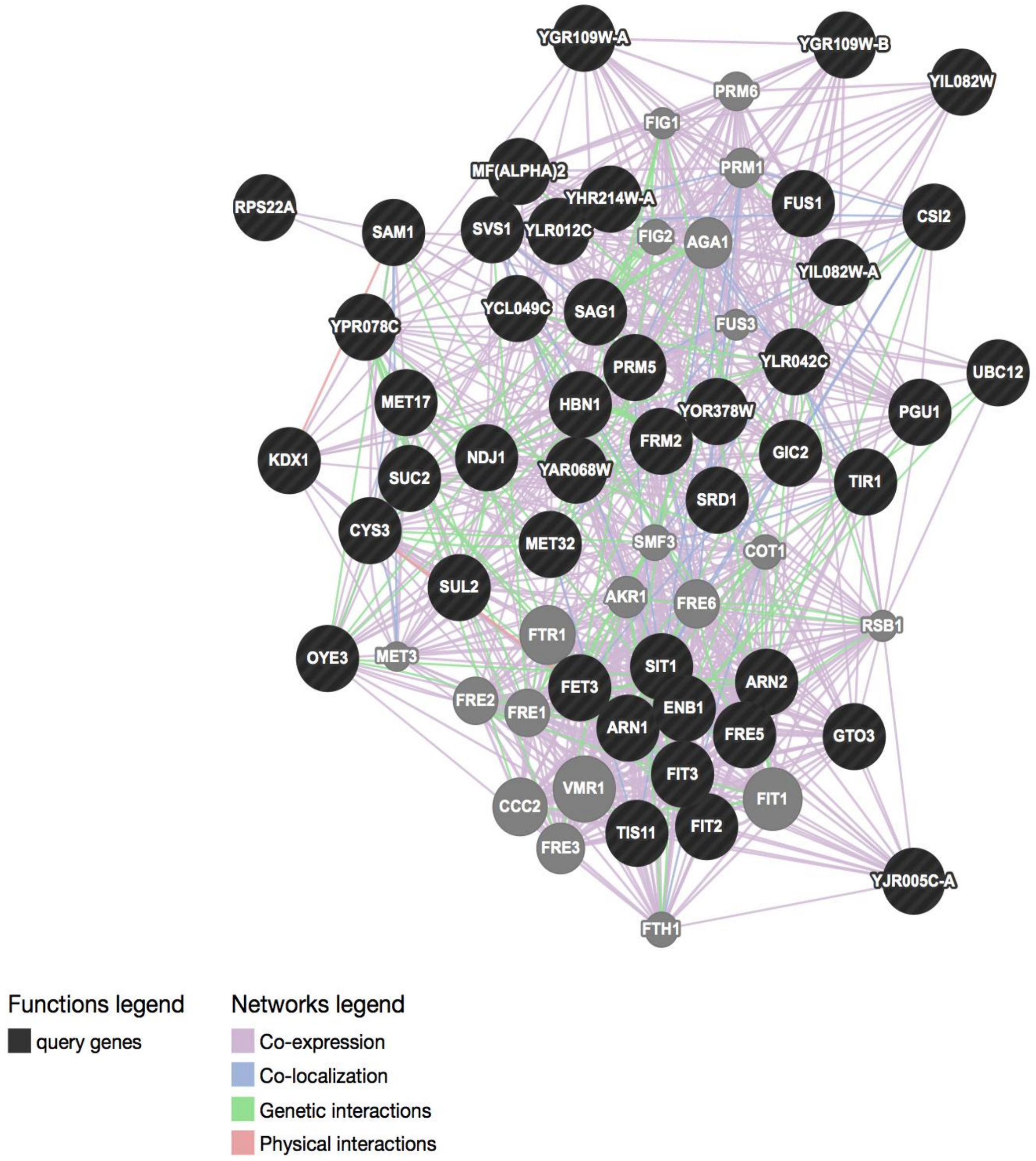

Figure S3. Network of interaction of up-regulated genes derived from microarray analysis (fixed thresholds of +2). Network analysis was performed using the GeneMANIA data service (Mostafavi *et al.*, 2008).

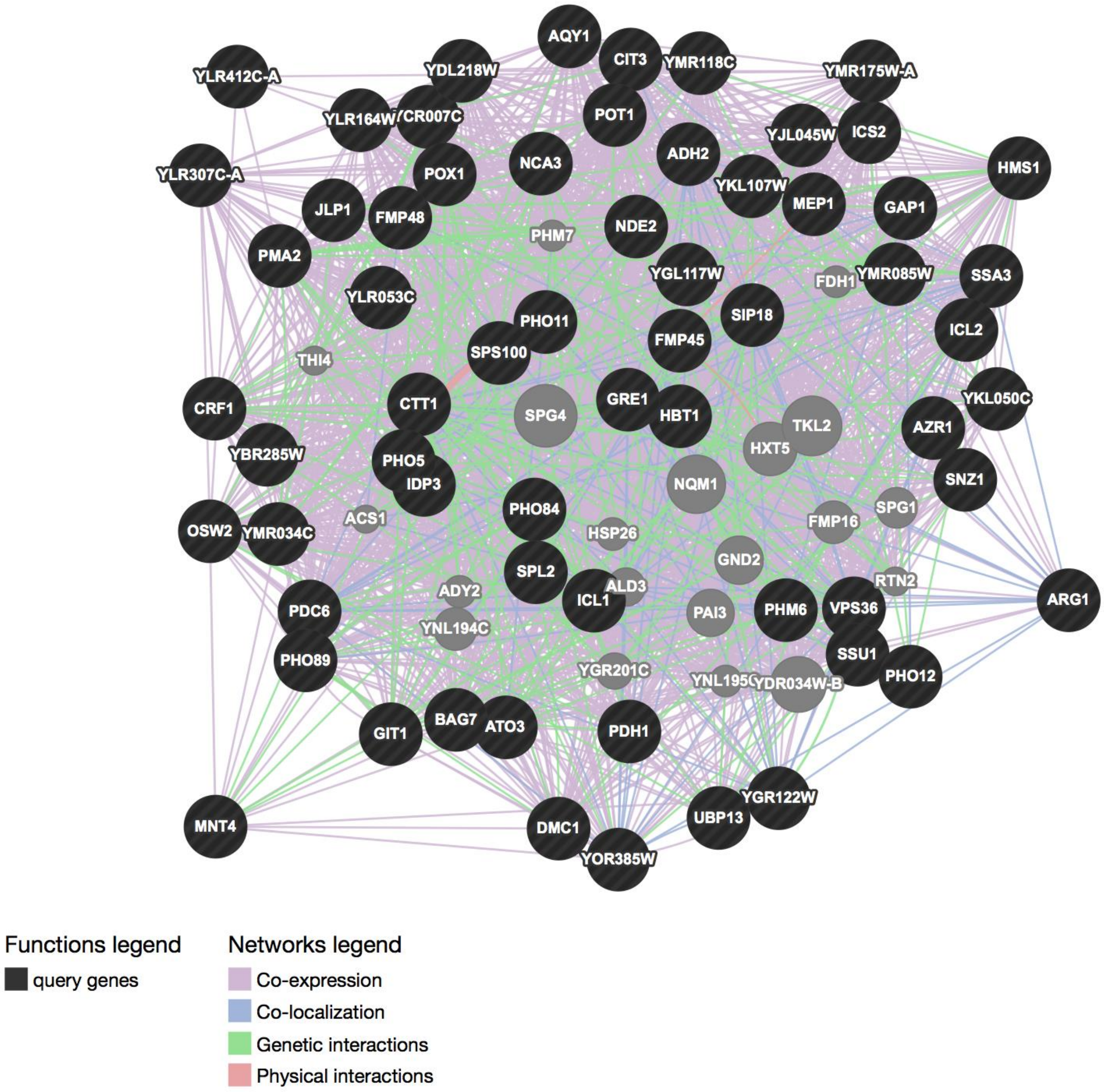

Figure S4. Network of interaction of down-regulated genes derived from microarray analysis (fixed thresholds of -2). Network analysis was performed using the GeneMANIA data service (Mostafavi *et al.*, 2008).

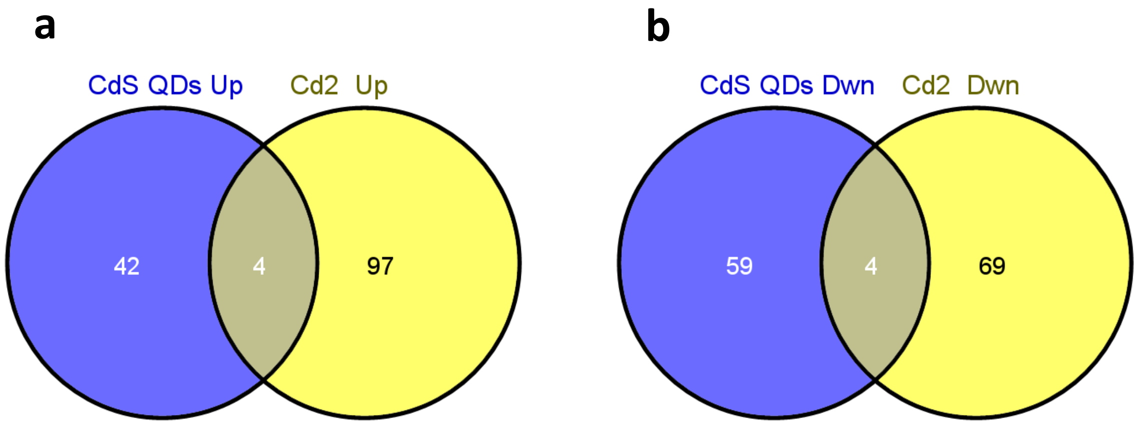

Figure S5. Venn's diagram representing the overlap between (a) up- and (b) down-regulated genes (fixed thresholds of +1;-1) with nystatin supplemented with CdS QDs and the data related Cd^{2+} ion response, from Jin *et al.* (2008).

References

1. Marmiroli M., Pagano L., Pasquali F. Zappettini A., Tosato V., Bruschi C.V., Marmiroli N. A genome-wide nanotoxicology screen of Saccharomyces cerevisiae mutants reveals the basis for cadmium sulphide quantum dot tolerance and sensitivity, *Nanotoxicology*, **2016**, *10* (1) 84–93.
2. Mostafavi S., Ray D., Warde-Farley D., Grouios C., Morris Q. GeneMANIA: a real-time multiple association network integration algorithm for predicting gene function. *Genom. Biol.*, **2008**, *9*, 1–15.

3. Jin Y.H., Dunlap P.E., McBride S.J., Al-Refai H., Bushel P.R., Freedman J.H. Global transcriptome and deletome profiles of yeast exposed to transition metals. *PLoS Genet*. **2008**, 4(4), e1000053.
